# Angiogenesis in Aortic Aneurysm and Dissection: A Literature Review

**DOI:** 10.31083/j.rcm2408223

**Published:** 2023-08-01

**Authors:** Yu Jia, Dongze Li, Jing Yu, Wenli Jiang, Yi Liu, Fanghui Li, Rui Zeng, Zhi Wan, Xiaoyang Liao

**Affiliations:** ^1^General Practice Ward/International Medical Center Ward, General Practice Medical Center, West China Hospital, Sichuan University, 610041 Chengdu, Sichuan, China; ^2^Department of Emergency Medicine and National Clinical Research Center for Geriatrics, Disaster Medicine Center, West China Hospital, Sichuan University West China School of Medicine, 610044 Chengdu, Sichuan, China; ^3^Institute of Biomedical Engineering, West China School of Basic Medical Sciences & Forensic Medicine, Sichuan University, 610041 Chengdu, Sichuan, China; ^4^Department of Cardiology, West China Hospital, Sichuan University, 610041 Chengdu, Sichuan, China

**Keywords:** aortic aneurysm, aortic dissection, angiogenesis, angiogenic factor, vasa vasorum

## Abstract

Aortic aneurysm and aortic dissection (AA/AD) are critical aortic diseases with a hidden onset and sudden rupture, usually resulting in an inevitable death. Several pro- and anti-angiogenic factors that induce new capillary formation in the existing blood vessels regulate angiogenesis. In addition, aortic disease mainly manifests as the proliferation and migration of endothelial cells of the adventitia vasa vasorum. An increasing number of studies have shown that angiogenesis is a characteristic change that may promote AA/AD occurrence, progression, and rupture. Furthermore, neocapillaries are leaky and highly susceptible to injury by cytotoxic agents, which promote extracellular matrix remodeling, facilitate inflammatory cell infiltration, and release coagulation factors and proteases within the wall. Mechanistically, inflammation, hypoxia, and angiogenic factor signaling play important roles in angiogenesis in AA/AD under the complex interaction of multiple cell types, such as smooth muscle cells, fibroblasts, macrophages, mast cells, and neutrophils. Therefore, based on current evidence, this review aims to discuss the manifestation, pathological role, and underlying mechanisms of angiogenesis involved in AA/AD, providing insights into the prevention and treatment of AA/AD.

## 1. Introduction

Aortic aneurysm and aortic dissection (AA/AD) are critical aortic diseases with a hidden onset and sudden rupture, which usually results in an inevitable death [1]. When the vessel wall cannot withstand the elevated blood pressure in the lumen, the aortic wall swells permanently, and pathological expansion exceeds 1.5 times the normal vascular diameter, forming an aortic aneurysm (AA). Alternatively, a local break occurs in the aortic intima, and the high-speed blood flow impact causes the intima/media to peel and expand, separating the middle layer of the arterial wall along the long axis, forming an aortic dissection (AD) [1]. Based on the anatomical location, AA is classified as thoracic AA (TAA), abdominal AA (AAA), or thoraco-abdominal AA. In contrast, AD can be categorized into Stanford type A (involving the ascending aorta) and type B (not involving the ascending aorta).

AA/AD have a prevalence of 1.3%–8% globally. Considering the increase in hypertension prevalence and population aging, AA/AD incidence continues to increase worldwide. A study from Sweden showed that AA/AD incidence increased by 52% (10.7–16.3 per 100,000 person-years) and 28% (7.1–9.1 per 100,000 person-years) in males and females, respectively, from 1987 to 2002 [2]. Although AD was previously considered a rare fatal disease, epidemiological data from Asia, such as China, showed that its incidence is as high as 2.78 per 100,000 person-years [3]. More importantly, there are limited effective targeted drugs for AA/AD with valid medical evidence. Surgery is currently the only effective therapeutic method for the clinical management of AA/AD. Therefore, it is imperative to strengthen our knowledge about the pathophysiology of aortic wall weakening and to discover comprehensive prevention and control strategies.

AA/AD development was closely related to disturbed vascular homeostasis, such as dysfunction of endothelial cells (ECs) and vascular smooth muscle cells (VSMCs), excessive inflammation, extracellular matrix (ECM) degradation, and angiogenesis [4]. Previous reviews have shown that EC dysfunction [5], VSMCs degeneration [6], and inflammatory cell infiltration [7] are essential in the pathophysiological changes of AA/AD. Notably, increasing studies have shown that angiogenesis is a characteristic change that promotes AA/AD occurrence, progression, and rupture by facilitating ECM remodeling and inflammatory cell infiltration. However, a more in-depth understanding of the pathological significance of angiogenesis in AA/AD will help researchers understand the pathological process of AA/AD from a new perspective to break through the current treatment dilemma.

## 2. The Concept, Types, and Models of Angiogenesis

Angiogenesis involves the growth of new capillaries from the existing blood vessels. It mainly manifests as the proliferation and migration of ECs of the adventitia vasa vasorum in aortic disease, extending to the tunica media and changing the local energy metabolism and tissue microenvironment [8]. In the AA/AD field, angiogenesis is a complex and dynamic interaction process between pro- and anti-angiogenic factors [9], relying on vascular ECs, VSMCs, fibroblasts, macrophages, mast cells, and ECM. However, angiogenesis-related diseases may develop and progress once this balance is disrupted. Recent studies have shown that pro-angiogenic factors mainly include the vascular endothelial growth factor (VEGF), matrix metalloproteinase (MMP), angiopoietin (ANGPT), platelet-derived growth factor, transforming growth factor (TGF), fibroblast growth factor (FGF), integrin, Wnt, and Notch families [10]. These factors play different and coordinated roles in the complex process of angiogenesis by interacting with the ECs and pericytes of neovascularization.

Sprouting angiogenesis is one of the most common angiogenesis mechanisms where sprouts are generated from the existing blood vessels and extend to form new blood vessels [11]. The function and distribution of tip and stalk cells have been identified as key factors in sprouting angiogenesis [9]. The tip cells are located in front of the blood vessels and stimulate angiogenesis in the microenvironment through their motile filopodia [12]. In contrast, the stalk cells are arranged behind the tip cells, proliferating rapidly to promote lumen formation [12]. In addition to sprouting angiogenesis, previous cancer-related studies have suggested intussusceptive angiogenesis, vessel co-option, vasculogenic mimicry, and vasculogenesis [13]. Sprouting angiogenesis was the default pathway of vessel formation in *ex vivo *angiogenesis assays, such as the aortic ring assay [14]; however, our understanding of the types of angiogenesis in aortic tissue is insufficient to date.

In exploring angiogenesis, many important models have been developed for studying its mechanisms. *In vivo* models include retinal formation, corneal microcapsules, sponge matrix glue, sponge implantation, and matrix glue suppository [10]. In contrast, vascular EC migration, proliferation, tubule formation, and aortic ring assay mainly comprise *in vitro* models [10]. These models are of great significance for exploring the pathophysiology of angiogenesis and for detecting the ability of anti- and pro-angiogenic factors to regulate angiogenesis, and almost all research methods included *in vitro* models for aortic disease.

## 3. Role of Angiogenesis in AA/AD

### 3.1 Structure of Normal Arteries

**Fig. 1.**
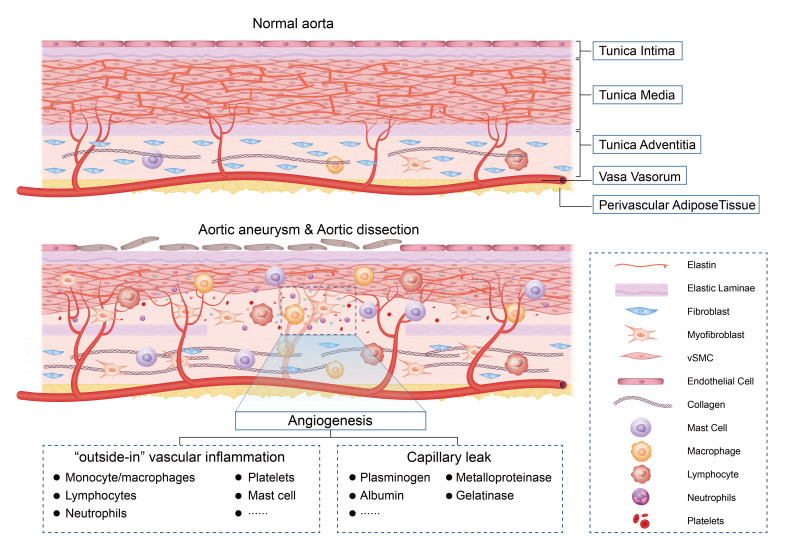
**Pathological role of angiogenesis in aortic aneurysm and dissection**. vSMC, vascular smooth muscle cell.

The aortic tissue of mammals mainly consists of intima, media, and adventitia (Fig. 1). The intima is located in the innermost layer, is in contact with blood flow, and consists of a monolayer of ECs and a subendothelial connective tissue layer limited by the internal elastic layer. The tunica media is located in the intermediate layer and is mainly composed of VSMCs and ECM components, such as proteoglycans, collagen, and elastin. The elastic lamina distinguishes the tunica media from the tunica intima and adventitia. In the normal arterial wall, the VSMCs and ECM components are arranged in an orderly manner and fill the tunica media to maintain the elasticity and strength of the aortic wall. The adventitia is the outer layer of the vessel, composed of fibroblasts and loose connective tissue, including the vasa vasorum. Aortic vasa vasorum vessels originate from the same or adjacent artery or vein, extend along the arterial wall, and penetrate the adventitia and two-thirds of the external tunica media, providing oxygen and nutrients [15].

### 3.2 Evidence of Angiogenesis in AA/AD Lesion Tissue

#### 3.2.1 Angiogenesis in Humans

In the normal aortic media, the vasa vasorum is sparse, and nutrition relies on diffusion from the lumen [16]. However, reports from 30 years ago have suggested an increased number of vasa vasorum associated with aneurysmal dilatation [17, 18, 19]. According to a report, medial microvessel density (MVD) was approximately 15-fold higher in AAA than in normal aorta [20]. In addition, the number of neovessels in patients with AAA was approximately three times higher than that in the atherosclerotic control group [20, 21]. Another study found that the density of CD34-positive microvessels was higher in AAA than in aortic occlusive disease [22]. Moreover, MVD was higher in inflammatory AAA than in atherosclerotic AAA [22]. Ultimately, angiogenesis is widely recognized as a characteristic change in AAA.

In the normal human thoracic aorta, the vasa vasorum is located between the outer third of the media and adventitia [8]. In TAA, neovascularization was increased close to the adventitia and extended inward across one-third of the external media [8, 23]. MVD (marked by von Willebrand factor (vWF)) in the media gradually decreases from the exterior to the interior, and these neovessels never reach the intima of the thoracic AA/AD [8]. Compared with healthy aortas, MVD was significantly higher in monogenic mutant and degenerative forms related to TAA than in bicuspid aortic valve (BAV) forms [8]. However, according to Billaud M* et al*. [24], adventitia MVD was decreased in patients with aneurysmal TAV compared with that in those with non-aneurysmal TAV. The different conclusions of these studies may be because of the different calculation methods used for MVD. In Billaud M’s study [24], MVD was calculated by dividing the total number of vessels observed on aortic cross-sections (hemotoxylin and eosin-stained) by the adventitial area. However, it is inaccurate to evaluate the neovessels by counting the number of blood vessels because the diameter of capillaries is usually ˂10 µm, thereby limiting distinguishing using light microscopy, and massive local infiltration of inflammatory cells may interfere with the counting.

In addition, previous studies have shown obvious thickening of the adventitial vasa vasorum in human thoracic AD [25, 26]. Vessel growth extended to the outer two-thirds and inner third of the media in 86% and 19% of thoracic AD samples, respectively [25]. Furthermore, vascular growth was observed in more than half of the samples at the edge of the thoracic AD [25]. Therefore, combined with experimental animal thoracic aortic samples, these results showed that angiogenesis occurs in abdominal aortic disease and thoracic AA/AD and that neovascularization penetrates the media originating from the adventitial vasa vasorum [23].

#### 3.2.2 Angiogenesis in an Animal Model

Animal models are an important way for researchers to explore disease mechanisms. Current animal models of AA/AD are primarily of rats and mice, and they are generally categorized into the following treatment categories: (1) extraluminal application of ${\mathrm{CaCl}}_{2}$ and elastase in the abdominal aorta to induce inflammation [27, 28]; (2) angiotensin-II (Ang II) subcutaneous infusion for ${\mathrm{ApoE}}^{-,/-}$ mice to mimic atherosclerosis-caused aortic dilatation and lesions; and (3) intraluminal application of elastase for the abdominal aorta or oral beta-aminopromazine (BAPN) to induce destruction of elastic layers followed by chronic inflammation [28, 29].

Although these models can induce the expansion of the aortic wall or dissection, none of them can completely simulate human AA/AD lesion characteristics [30]. For example, the intraluminal rather than the extraluminal application of elastase-induced AAA is complicated by media angiogenesis, which is a common characteristic of AA/AD [28]. Furthermore, angiogenesis in the human AA/AD sample was more dominant than that in the animal AA/AD model [20]. This phenomenon may be explained by several reasons as follows: AA/AD in humans is usually caused by decades of damage to vessels, but animal models were induced in ˂1 month; application of ${\mathrm{CaCl}}_{2}$ induces AA and results in the absence of MMP2 and MMP9, but they are abundant in human AA and have a dramatic pro-angiogenic effect; and mural thrombosis is common in human AA/AD, and the thrombus-covered wall is hypoxic, particularly in the inner thirds of the media, but all of the above-accepted models are without an obvious mural thrombus [31]. Moreover, angiogenesis was observed in angiotensin II- [32, 33], ${\mathrm{CaCl}}_{2}$- [34], elastase- [35], and BAPN- [36] induced AAA models, which indirectly verified that angiogenesis in the media and adventitia is one of the pathological features of AAA. However, evidence of angiogenesis in abdominal or Stanford type B-AD is lacking to our knowledge. Furthermore, this phenomenon may be due to insufficient attention given to type B-AD in preclinical studies.

### 3.3 The Pathological Role of Angiogenesis in AA/AD

#### 3.3.1 Association between Angiogenesis and AA/AD Progression

Angiogenesis is associated with AA/AD incidence and disease severity. For example, the upregulation of α_v_mRNA and α_v_β3 integrin in the blood vessels surrounded by a matrix-expressing tenascin suggests that angiogenesis is an ongoing process in mature AA [37]. Furthermore, Choke E *et al*. [22] reported that human medial neovascularization is increased in the rupture edge of AAA than in the non-ruptured aneurysm anterior sac. Similarly, MVD is enhanced in patients with ruptured AAA than in those with non-ruptured AAA [21]. Moreover, increased neovessel growth was observed at the edge of type A-AD [25]. These results have attracted more attention to the pathological role of angiogenesis in AA/AD.

#### 3.3.2 Association between Angiogenesis and “Outside-In” Vascular Inflammation

Traditional concepts of vascular inflammation are considered ‘inside-out’ responses centered on the monocyte adhesion and lipid oxidation hypotheses [38, 39]. In AA/AD, the adventitia changes significantly during angiopathy progression and thickens with the expansion of the vascular wall [40]. Adventitial vasa vasorum neovascularization is associated with a marked increase in capillary permeability [41] and chemokine levels [42], which may enhance the migration of inflammatory cells to angiogenic sites. Therefore, adventitial cells become highly populated by macrophages, lymphocytes, and neutrophils (Fig. 1) [40]. Indeed, AAA pathogenesis initiates macrophage migration into the adventitia, followed by a subsequent presentation in the media [43]. Therefore, according to the “outside-in” hypothesis, vascular inflammation of AA/AD is initiated from the adventitia neovessel and progresses inward toward the intima [44]. This explains that the vascular pathology of AA/AD (outside-in), to some extent, involves stronger pathological changes, such as media degradation and angiogenesis, than that of atherosclerotic angiopathy (inside-out).

#### 3.3.3 Angiogenesis Promotes Inflammatory Infiltration

Numerous evidence has demonstrated that macrophages are involved in AA/AD by secreting pro-inflammatory factors, metalloproteinases, and other substances to induce VSMCs apoptosis, ECM degradation, and neovessel formation, leading to aortic wall destruction and weakening [45, 46]. Most macrophages that accumulate in the aneurysmal or dissected aortic wall originate from circulating monocytes, whereas only a few macrophages are derived from aortic tissue-resident macrophages.

Previous studies revealed a close spatial correlation between neovessels and inflammatory infiltration in the AAA wall, of which most cells were monocytes/macrophages and lymphocytes [20]. Moreover, neovascularization was positively correlated with the number of lymphocytes in AAA samples (CD31: r = 0.625; CD105: r = 0.692) [47]. The predominant lymphocyte cell infiltrates were shown as ${\mathrm{CD4}}^{+}$${\mathrm{CD8}}^{+}$T cells and infiltrates of type 2 Th cells, and their production induces AAA [48]. Kokje VBC *et al*. [49] reported that neutrophils infiltrated extensively into AAA tissues and had positive staining (myeloperoxidase staining) in the proliferative vasa vasorum, whereas they were absent in atherosclerotic control samples. This result suggests that angiogenesis promotes neutrophil infiltration, which has been proven to induce the occurrence and development of AA/AD [50, 51, 52]. Furthermore, the number of microvessels identified by CD31 was markedly increased in the human and mouse AAA models, consistent with harmful platelet infiltration [53]. These results suggest that neovascularization in all arterial wall layers is prominent, and angiogenesis can facilitate chronic inflammation [20].

A previous study confirmed lymphangiogenesis in the AAA wall [47], and lymph stasis was observed using indocyanine green fluorescence lymphography. Additional results demonstrate that enhanced infiltration by angiogenesis and relatively insufficient lymph drainage are associated with an increased number of macrophages in AAA [54].

#### 3.3.4 Angiogenesis Promotes Protein and Enzyme Infiltration

Excluding the infiltration of immune-inflammatory cells caused by poor mural cell coverage and defective endothelial junctions, Kessler K *et al*. [8] found that an incomplete endothelial structure was associated with plasminogen and albumin accumulation in the media of human aorta samples, leading to TAA remodeling and weakening. Moreover, tissue inhibitors of metalloproteinase and gelatinase (collagenase) are localized to the vasa vasorum of AA, which strongly suggests that angiogenesis possibly involves the genesis of AA/AD [18].

## 4. The Mechanisms and Target of Angiogenesis in AA/AD

**Fig. 2.**
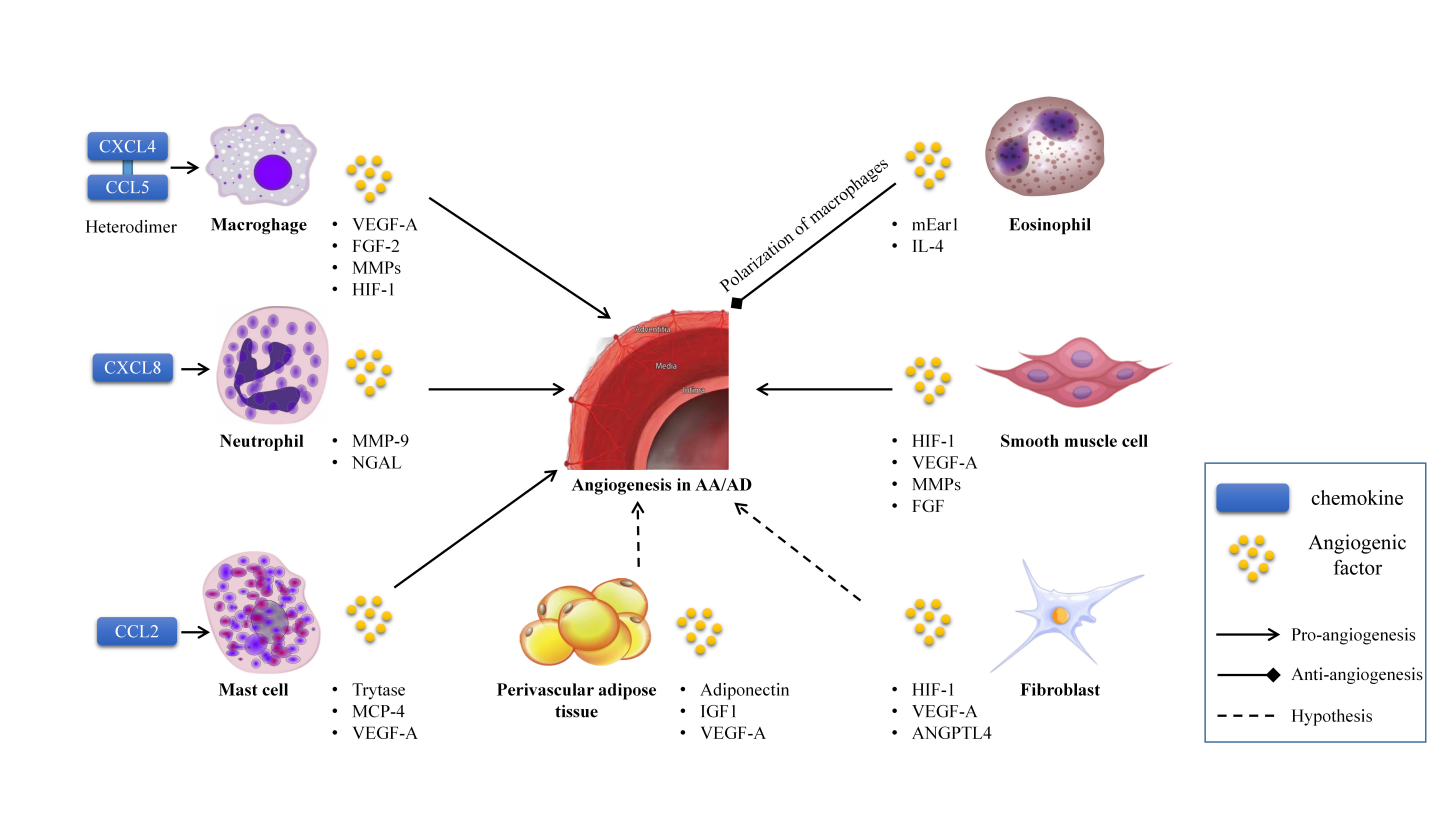
**The interplay of cells, chemokines, and angiogenesis in aortic aneurysm and dissection**. VEGF, vascular endothelial growth factor; HIF-1, hypoxia-inducible factor 1; IL-4, interleukin-4; FGF-2, fibroblast growth factor 2; MMPs, matrix metalloproteinases; NGAL, neutrophil gelatinase-associated lipocalin; MCP-4, mast cell protease-4; IGF1, insulin-like growth factor 1; ANGPTL4, angiopoietin-like 4; AA/AD, aortic aneurysm and aortic dissection; CXCL, C-X-C motif chemokine ligand; CCL, C-C motif chemokine ligand. Cell pictures come from the internet: https://699pic.com/tupian/598139.html. This network image supports all-purpose authorization.

**Fig. 3.**
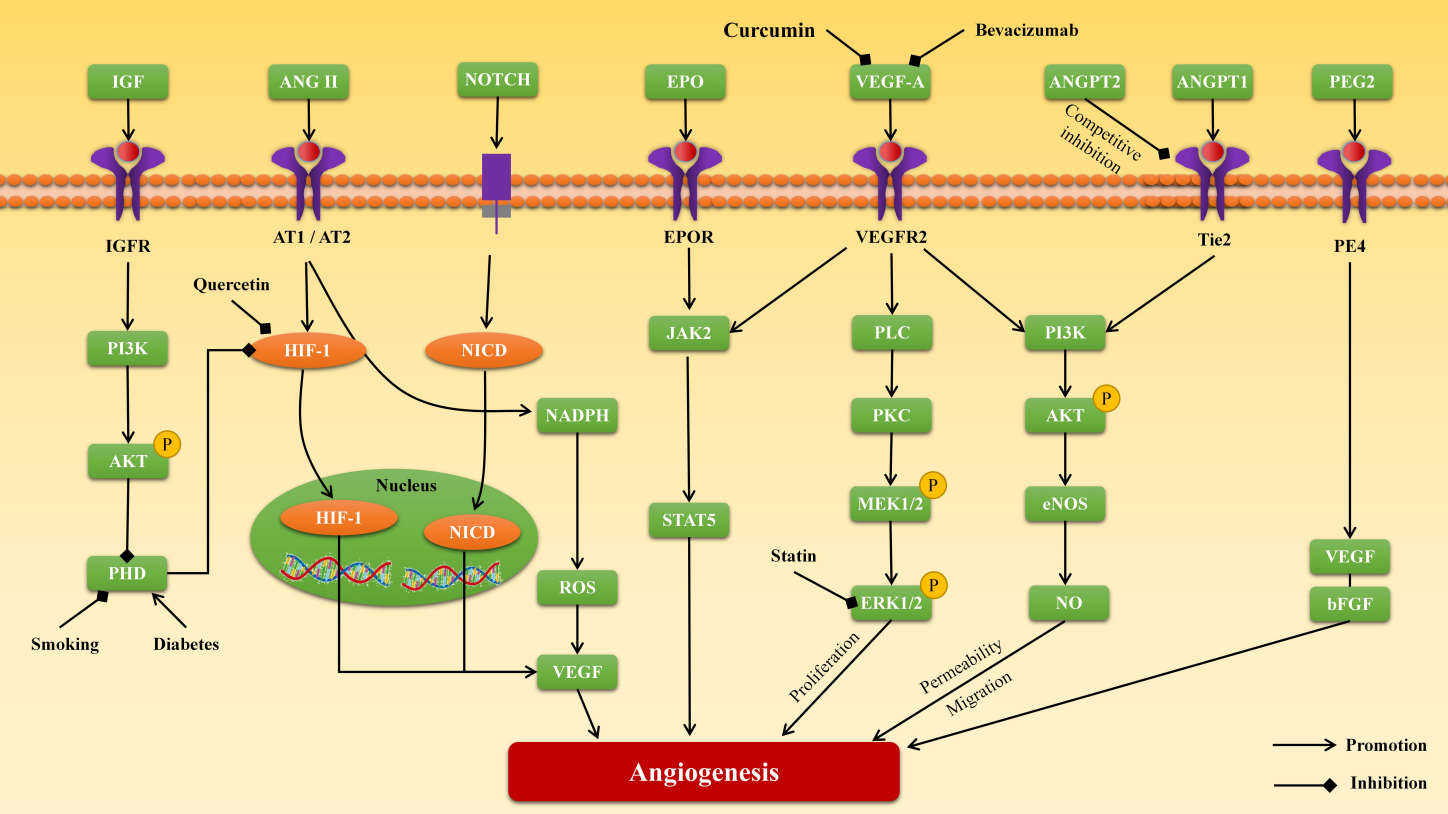
**The mechanisms and targets of angiogenesis in aortic aneurysm and dissection**. VEGF, vascular endothelial growth factor; HIF-1, hypoxia-inducible factor 1; PHD, prolyl hydroxylase; FGF, fibroblast growth factor; IGF, insulin-like growth factor; ANGPT, angiopoietin; ANG II, angiotensin II; AT1, angiotensin type 1 receptor; AT2, angiotensin type 2 receptor; EPO, erythropoietin; PGE2, prostaglandin E2; PLC, phosphoinositide-phospholipase C; PKC, protein kinase C; ROS, reactive oxygen species; PI3K, phosphatidylinositol-3-kinase; NICD, notch intracellular domain; AKT, protein kinase B (PKB); JAK2, Janus kinase 2; NADPH, nicotinamide adenine dinucleotide phosphate hydrogen; STAT5, signal transducer and activator of transcription 5; ERK, extracellular signal-regulated kinase; MEK, mitogen-activated extracellular signal-regulated kinase; eNOS, endothelial nitric oxide synthase.

In the aortic remodeling field, local chronic inflammation and relative hypoxia are the main triggers of angiogenesis in the media and adventitia, mediated through several angiogenic factors, such as VEGF, MMP, ANGPT, hypoxia-inducible factor (HIF), and fibroblast growth factor (FGF) (Fig. 2). Anti-angiogenic treatment has been proven effective in reducing AA/AD incidence and progression in various animal models, suggesting the potential of angiogenesis-targeted therapy for AA/AD (Fig. 3).

### 4.1 Immunity and Inflammation 

We discussed above that neovascularization promotes inflammatory infiltration, such as of macrophages, lymphocytes, mast cells, and eosinophils (Fig. 2). These inflammatory cells also proved to be closely related to angiogenesis by releasing pro-inflammatory factors, matrix protease, and chemokines to induce pro-angiogenic effects.

#### 4.1.1 Macrophages and Chemokines

Numerous studies have confirmed that macrophages are the main inducers of aortic vascular inflammation and play a key role in AA/AD progression [45, 46]. In addition to their immunological and inflammatory functions, they promote angiogenesis by producing pro-angiogenic cytokines and growth factors, such as VEGF-A and basic FGF-2, in animal models [36]. Moreover, in patients with Stanford type A-AD, macrophages play a central role in aortic wall remodeling by inducing the release of MMPs and pro-inflammatory cytokines and promoting excessive angiogenesis [25].

C-X-C motif chemokine ligand (CXCL) 4, which is a platelet-derived chemokine, and CCL5 (RANTES) are both monocyte-attracting chemokines [55]. They can form a C-type CXCL4/CCL5 heterodimer that enhances CCL5-induced monocyte arrest, adhesion, and migration [56]. MKEY is a peptide inhibitor of CXCL4-CCL5 heterodimer formation that attenuates aortic diameter enlargement by inhibiting mural macrophage infiltration and angiogenesis [53]. Therefore, inhibiting macrophage functions and aggregation may mitigate AA/AD progression by improving angiogenesis.

#### 4.1.2 Mast Cells

Mast cells possess multiple biological functions, including innate immunity, participation in host defense mechanisms against parasitic infections, regulation of the immune system, tissue repair, and angiogenesis [57]. In human AAA samples, the number of infiltrated mast cells increased in the adventitia and outer media [34]; moreover, a study found that T cell activation, elastin levels, and angiogenesis were inhibited in mast cell-deficient mutant white spotting/white spotting (Ws/Ws) rats modeled by periaortic application of ${\mathrm{CaCl}}_{2}$ treatment [34]. Inhibition of mast cell degranulation plays a similar protective role against AAA [34]. This study suggests that mast cells and their granzymes are important inducers of angiogenesis and AAA. Furthermore, mouse mast cell protease-4 (mMCP-4) is a key trigger of angiogenesis and vascular cell apoptosis in AAA [58]. Mechanistically, mMCP-4 secreted by mast cells promotes aortic ring microvessel outgrowth via VEGF synthesis by ECs in a mouse aortic ring assay [58]. Therefore, the study emphasizes the critical role of mast cell chymase in AAA. Moreover, Zhang *et al*. [59] revealed that chemokine (C-C motif) receptor 2 (CCR2) is the key chemokine receptor for mast cell recruitment in Ang II-induced AAA lesions. Similarly, CCR2 knockout in mast cells protected from AAA by decreasing angiogenesis, macrophage and T cell infiltrations, and medial smooth muscle cell loss [59].

#### 4.1.3 Eosinophils

Eosinophils are innate immune cells that are rich in inflammatory cytokines, chemokines, and growth factors [60]. A clinical study suggested that eosinophil count is an independent risk factor for AAA incidence [61]. However, eosinophil gene knockout exacerbated AAA growth with increased inflammatory infiltration and angiogenesis in Ang II-induced ${\mathrm{Apoe}}^{-,/-}$ mice [61]. Mechanistically, eosinophil-derived mouse eosinophil-associated-ribonuclease-1 and interleukin (IL)-4 trigger the polarization of macrophages from M1 to M2, resulting in ECM remodeling and wall weakening [61].

#### 4.1.4 Inflammatory Factors and Receptors

Type I interferons (IFNs) are cytokines commonly produced by immune cells following exposure to antigenic stimuli, including bacteria, viruses, autoantigens, and tumors [62]. IFN signaling through IFN receptors (IFNRs) initiates the immune-inflammatory reactions [62]. Compared with wild-type mice, IFNAR1 knockout mice showed mitigated neoangiogenesis, leukocyte accumulation, and time-dependent infrarenal aortic enlargement [63]. Therefore, IFNs are key cytokines in immune inflammation that are also associated with angiogenesis in AA/AD.

Kallistatin is a member of the serine proteinase inhibitor (SERPIN) family, which is associated with anti-inflammation, anti-oxidative stress, and anti-angiogenesis [64]. Treatment with recombinant human kallistatin or transgenic overexpression of the human kallistatin gene limited AAA progression in Ang II- and calcium phosphate-induced mouse models [65]. Although this study does not directly show that kallistatin can inhibit angiogenesis, the gene expression of VEGF in transgenic mice overexpressing the human kallistatin gene was lower than that in wild-type mice as well as in cultured VSMCs. Therefore, kallistatin might protect against AA/AD through anti-angiogenesis; however, this should be validated using an *in vitro* angiogenesis assay.

Similar to MMPs, cysteine cathepsins are commonly found in lysosomes, where they are involved in intra- and extracellular protein degradation [66]. Qin *et al*. [32] found that elastolytic cathepsin S gene knockout significantly reduced lesion adventitia microvessel content, inflammatory cell infiltration, and AAA formation in an Ang II infusion-induced mouse model. These results suggest that the degradation of ECM by proteases is closely related to angiogenesis.

### 4.2 Hypoxia Signaling Pathway

#### 4.2.1 Hypoxia-Inducible Factor 1 

HIF-1 consists of an oxygen-regulated α subunit and a constitutively expressed β subunit, which regulate cell adaptation to hypoxia, such as migration, proliferation, survival, and angiogenesis [35]. Intervention with HIF-1α inhibitors limited angiogenesis, leukocyte infiltration, and aneurysm progression in an elastase-induced AAA mouse model. Similarly, another study showed that silencing of the *HIF-1α* gene alleviated aneurysm enlargement, angiogenesis, and expression of pro-angiogenic and pro-inflammatory factors [67], such as VEGF, Flt-1, MMP-2, and MMP-9, in an Ang II-infused AAA model. In addition, deferoxamine, which is a prolyl hydroxylase inhibitor, stabilizes HIF-1α, augments MMP activities, and exacerbates the severity of Ang II-induced AAA [68].

Moreover, macrophage HIF-1α activation triggers vascular inflammation and AD progression by targeting metallopeptidase domain 17 (ADAM17) [36]. Besides macrophages, HIF-1 signaling in VSMCs also plays an essential role in angiogenesis [69]. Wang *et al*. [69] reported that cyclooxygenase-2 (COX-2) upregulated HIF-1α/VEGF signaling, leading to angiogenesis and AAA formation in a ${\mathrm{CaCl}}_{2}$-induced mouse model; however, this biological process could be inhibited by quercetin, a flavonoid extracted naturally and has been validated to inhibit AAA progression by limiting oxidative stress and inflammatory response. Furthermore, a study included gene sequencing using microarrays for the emergency repair of ruptured AAA and open elective repair of AAA [70]. The upregulated genes, such as *ANGPTL4*, *HILPDA*, *LOX*, and *SRPX2*, involved in the processes of canonical HIF-1α signaling pathway network-related angiogenesis, are highly expressed in fibroblasts rather than in macrophages and VSMCs [70]. Previous studies have focused on the important roles of inflammatory cells and VSMCs in AA occurrence and development. Fibroblasts, which are the main cell component of the adventitia, are important in maintaining the stability of the arterial structure and function [71]. However, the pathological mechanisms of fibroblasts in AA/AD have rarely been reported. Therefore, angiogenesis may be a critical mechanism for exploring the role of fibroblasts in AA/AD. Taken together, HIF-1 is a potential target for hypoxia signaling pathway-related angiogenesis in AA/AD (Fig. 3).

#### 4.2.2 Diabetes 

The risk factors for AA/AD are similar to those for cardiovascular diseases; however, diabetes is an exception. Several studies have indicated that AA/AD risk is lower in patients with type II (insulin-resistant) diabetes than in healthy controls [72, 73, 74]. Therefore, researchers have focused on how diabetes inhibits AA/AD progression. Guo *et al*. [75] found an explanation that the pharmacological inhibition of prolyl hydroxylase reversed the impairment of HIF-1 expression and activity in diabetes and obviously counteracted the suppression of AAA enlargement by diabetes, with increased angiogenesis, leukocyte infiltration, and medial elastin and VSMCs destruction. Therefore, patients with diabetes have a lower risk, and the severity of AA/AD may result, at least partly, from dysregulated HIF-1-associated angiogenesis.

#### 4.2.3 Smoking 

Cigarette smoking is the most dangerous environmental risk factor for AA/AD incidence and progression [76], and smoking individuals have a 2.5-fold greater risk for AAA than for atherosclerosis [77]. In AAA (n = 75) and control (n = 11) samples, all tissues exhibited increased angiogenesis-related gene expression and signs of oxidative stress during active smoking [78]. Several studies have shown that cigarette smoke or its extract inhibits prolyl hydroxylase, resulting in HIF-1 activation [79, 80], which may be a potential mechanism of angiogenesis induced by smoking. Moreover, cigarette smoke induces oxidative stress and reactive oxygen species, which in turn induces angiogenesis through HIF-1 [81]. Therefore, smoking leads to AA/AD involving angiogenesis through the hypoxia signaling pathway.

#### 4.2.4 Erythropoietin

Chronic anemia and hypoxia are the principal inducers of production of erythropoietin (EPO), which is a critical cytokine regulating erythropoiesis and is synthesized in the kidneys [82]. Treatment with EPO monoclonal antibodies and EPO receptor (EPOR) gene knockdown (${\mathrm{Epor}}^{+,/-}$${\mathrm{Apoe}}^{-,/-}$) significantly reduced the incidence of AAA in an Ang II-induced mouse model [83]. Mechanistically, EPO induced endothelial migration, proliferation, and tube formation through the JAK2/STAT5 signaling pathway *in vitro* and *ex vivo* experiments [83]. Therefore, it is suggested that the hypoxia signaling pathway induces angiogenesis and may participate in the progression of AA/AD development.

#### 4.2.5 PI3K/AKT

Phosphatidylinositol-3-kinase (PI3K) signaling has multiple biological functions, such as cell differentiation, motility, survival, proliferation, and growth [84]. Previous studies have suggested that pan-PI3K inhibition leads to decreased levels of HIF-1α, macrophage infiltration, and aneurysm dilatation in the aortas of porcine pancreatic elastase-infused rats [85]. However, systemic inhibition of pan-PI3K is associated with severe side effects, such as hepatotoxicity, stomatitis, pneumonitis, bone marrow suppression, hyperlipidemia, and hyperglycemia [86]. Furthermore, a recent study reported that treatment with IPI-549, which is a specific PI3K inhibitor, significantly inhibited angiogenesis and immune cell infiltration and prevented AAA formation in elastase-infused mice [85]. Mechanistically, IPI-549 treatment decreased AKT phosphorylation and HIF-1α levels; therefore, the PI3K/pAKT/HIF-1α signaling pathway is considered to play an essential role in AAA [85].

### 4.3 Angiogenic Factors and Signaling Pathways

#### 4.3.1 VEGF and vascular endothelial growth factor receptor (VEGFR) 

The VEGF family consists of five homologous genes, including *VEGF-A*, *VEGF-B*, *VEGF-V*, *VEGF-D*, and *PGF * [87], all of which are associated with angiogenesis and lymphangiogenesis. VEGF-A is an EC growth factor with a highly conserved cystine domain, heparin-binding site, and secreted signal peptide, which specifically binds to VEGFRs to play a biological role. The angiogenic effect of VEGF-A is mediated by VEGFR2 (Flt-1) and upregulated by inflammation, hypoxia, oxidative stress, wound healing, and other factors through transcriptional regulation mediated by various transcription factors, including HIF-1 (Fig. 3) [88, 89]. VEGF-A is one of the most potent angiogenic and vascular permeability factors that are essential in angiogenesis throughout life and are required for embryonic development [90, 91].

Kaneko H *et al*. [33] demonstrated that VEGF-A/Flt-1 signaling is important in ${\mathrm{CaCl}}_{2}$-induced AAA development by affecting both neovascularization and chronic inflammation. Injection with soluble Flt-1 (competitive inhibition of Flt-1) inhibited the infiltration of inflammatory cells, MMP activity, ECM degradation, and angiogenesis and finally alleviated AAA expansion [33]. Currently, the anti-VEGF-A monoclonal antibody, bevacizumab, is approved as an effective adjunctive therapy for solid tumors [92]. However, severe hypertension is a common adverse effect of bevacizumab [93]. Regardless of whether Flt-1 or bevacizumab is used, local rather than systemic medical treatment may be more suitable for future clinical trials. In addition to the biosynthetic drugs, curcumin, purified from the roots of *Curcuma longa*, was shown to inhibit VEGF expression and angiogenesis in the ${\mathrm{CaCl}}_{2}$-induced TAA model [23]. Therefore, curcumin may be a potential intervention in angiogenesis during AA/AD progression.

#### 4.3.2 Notch Pathway

Notch activity is important for cell differentiation and involves angiogenesis by controlling the conversion of tip/stalk ECs [9]. The Notch pathway inhibits VEGFR2, VEGFR3, and NRP1 expression and enhances VEGFR1 expression [94]. The main function of VEGFR1 is to competitively bind to VEGF and inhibit VEGFR2 to regulate VEGF signaling in ECs [95]. The Notch intracellular domain (NICD) receptors depend on proteolytic cleavage by γ-secretase [96]. In AAA, dibenzazepine, which is a γ-secretase inhibitor, prevents Ang II-induced angiogenesis by inhibiting VEGF/VEGFR and HIF-1α expression [97]. Therefore, intervention in the Notch pathway appears to involve multiple mechanisms of vascular protection, including inflammation and angiogenesis [96].

#### 4.3.3 Angiopoietin and Tie2 Receptor

The ANGPT system and its Tie2 receptor are related to the integrity and stability of the blood vessels [98]. ANGPT2 inhibits ANGPT1 by competitively binding to Tie2; therefore, ANGPT2 acts as an antagonist of ANGPT1 for Tie2 interaction. According to Yu’s report, recombinant ANGPT2 administration significantly inhibited angiogenesis, monocyte/macrophage infiltration, aortic dilatation, and rupture in an Ang II-induced ${\mathrm{ApoE}}^{-,/-}$ mouse AA model [99]. Similarly, Chen *et al*. [100] analyzed the DNA methylation patterns of type A-AD and controls to explore epigenetic changes during AD progression. They found that DNA methylation of the *ANGPT2* gene was lower in the AD testing and verification samples [100]. AD has a higher transcriptional activity of ANGPT2 because the hypermethylation of DNA leads to gene silencing. Finally, gene ontology (GO) analysis showed that angiogenesis was the most significant biological process [100]. Therefore, these results suggest that DNA methylation of *ANGPT2* leads to angiogenesis and AD formation.

However, it has been suggested in atherosclerosis that ANGPT1/Tie2 play anti-angiogenic, anti-inflammatory, and anti-atherogenic roles [101, 102]. Several studies have revealed that the functions of ANGPT1 and ANGPT2 in atherosclerosis are complex and paradoxical [103, 104, 105, 106]. Therefore, the roles of these two molecules might involve functions other than antagonism.

#### 4.3.4 Prostaglandin E2

Prostaglandin (PG) E2 is the most abundantly detected PG in various tissues biosynthesized by cyclooxygenase-1 (COX-1) or COX-2 and has four receptor subtypes (EP1-4). PGE2 is widely accepted to induce angiogenesis and inflammation in cancer and vascular diseases [107, 108]. COX-2 and the microsomal isoform of PGE synthase (mPGES-1) are involved in PGE2 synthesis, which is associated with vascular lesions in AAA [109]. Furthermore, a study demonstrated that PGE2 directly induces angiogenesis through EP4 in an *in vitro* angiogenesis assay [109]. Therefore, the COX-2/mPGES-1/PGE2/EP4 axis may be a potential intervention target for AAA-associated hypervascularization.

#### 4.3.5 CXCL8 

CXCL8, known as IL-8, is considered a pro-angiogenic factor that promotes chemotaxis and proliferation of ECs [110]; however, it has strong chemotactic effects on immune cells, particularly neutrophils. Therefore, CXCL8 can indirectly induce angiogenesis through chronic inflammation [111]. Blocking CXCL8 signaling by CXCR1/CXCR2 inhibitors (DF2156A) preserves the integrity of the vessel wall and inhibits leukocyte infiltration through the vasa vasorum [49].

#### 4.3.6 Matrix Metalloproteinases 

MMPs belong to a family of proteolytic enzymes that degrade several components of the ECM. The pathological role of the MMP family in AA/AD, including MMP-1, -2, -3, -9, -12, -13, and -14, has been widely accepted [112, 113]. Of these MMPs, MMP-9 has been validated as a pro-angiogenic factor in AAA. However, lentiviral-mediated silencing of MMP-9 through RNA interference in human ECs failed to induce migration, proliferation, and tube formation in Matrigel matrix [114]. Therefore, MMP-9 regulates vascular remodeling by degrading ECM and promoting angiogenesis in AA/AD.

#### 4.3.7 Plastin-3

A previous study showed that aortic inducible nitric oxide synthase (iNOS) promotes NO expression, which plays a critical role in Ang II-induced AA and Marfan syndrome models [115]. A recent study showed that endothelial S-nitrosylation (SNO) modification promotes the development of thoracic aortic dissection (TAD) through plastin-3 (PLS3) SNO modification [116]. PLS3 SNO increased the production of the PLS3/plectin/cofilin complex, which enhanced cell migration and tube formation in an Ang II-treated EC angiogenesis assay [116]. Therefore, these results suggest that iNOS-generated NO promotes pathological angiogenesis and TAD formation by modifying endothelial PLS3 through SNO.

### 4.4 Non-Coding RNA

Non-coding RNA (ncRNA) has gradually been recognized as a type of RNA that does not encode proteins but affects the stability or function of mRNA through post-transcriptional regulation [117]. Li *et al*. [118] analyzed human TAD by analyzing the microarray profiles of long ncRNA (lncRNA). They found that lncRNAs with significant differential expression (fold change >4.0, *p* < 0.01) were associated with angiogenesis [118]. BTG1, HIF-1A, and RUNX1, which positively regulate angiogenesis, have been shown to interact with lncRNAs RP11-796E2.4, HIF-1A-AS2, and AX746823 [118]. Therefore, these lncRNAs may be potential targets for treating AA/AD through anti-angiogenesis.

Similar to lncRNAs, microRNAs (miRNAs) are small ncRNAs. Sun *et al*. [119] reviewed miRNAs in angiogenesis-related diseases and particularly summarized that angiogenesis-related miRNAs are involved in AA/AD, such as miR-29, miR-25, and miR-155. These miRNAs were upregulated or downregulated in the plasma or tissue, whereas some were validated to play a critical role in AA/AD progression [120, 121]. However, to the best of our knowledge, no ncRNA directly regulates angiogenesis in AA/AD. Therefore, some potential ncRNAs require further exploration.

### 4.5 Intervention Drugs and Signaling Pathways

Epidemiological studies with large sample sizes have shown that hypercholesterolemia is associated with modest AAA risk (odds ratio: 1.31–1.44) [122]. Although significant heterogeneity existed, one meta-analysis demonstrated that statin therapy effectively reduced the risk of AAA growth rates and mortality [123]. However, preclinical studies showed that AAA vascular lesions in hypercholesterolemic and normal control mice were comparable with those of elastase-induced AAA, and no differences were found in inflammatory infiltration and mural angiogenesis between spontaneous hyper- and normo-cholesterolemic mice [124]. Moreover, many studies have found that statin treatment can inhibit angiogenesis [125]. Therefore, anti-angiogenesis, rather than lipid-lowering effects, could be the potential mechanism of statins in AAA treatment.

Zhang *et al*. [126] revealed that simvastatin ameliorates AAA formation in Ang II-induced ${\mathrm{ApoE}}^{-,/-}$ mice. Mechanistically, simvastatin inhibited Ang II-induced tube formation and MMP-2 released by human umbilical vein ECs, at least partly via EKR signaling pathways in a Matrigel assay [126]. Furthermore, Escudero *et al*. [127] conducted preclinical studies by combining already available clinical drugs to enhance the anti-angiogenic ability and reduce side effects. They considered that bexarotene has an anti-angiogenic activity but could lead to dyslipidemia, whereas some clinical studies suggested that statins counteract this adverse effect [128]. Moreover, bexarotene is an RXRα high-affinity synthetic ligand, and statins can interact with peroxisome proliferator-activated receptors (PPARs). PPARs and their heterodimer complexes with RXRα synergistically respond to the agonists of RXR [128]. Finally, the authors demonstrated that rosuvastatin combined with bexarotene reduced AAA formation, inflammation, and neovascularization compared with their single treatment and the blank control [128]. Furthermore, combined therapy inhibited EC vascularization, sprouting, and the release of angiogenic factors in an *in vivo *Matrigel assay and *ex vivo* murine aortic ring assay [127]. Mechanistically, the anti-angiogenesis effect was caused by the inhibition of Ang II-induced activation of the Akt/mTOR/P70S6K1 signaling pathway [127].

Heat shock protein 90 (HSP90) is a conserved molecular chaperone that is involved in many biological processes, including cell proliferation, migration, and survival under normal and stressful conditions [129]. In addition, 17-dimethylaminoethylamino-17-demethoxygeldanamycin (17-DMAG), which is a semi-synthetic derivative of geldanamycin, is an inhibitor of HSP90 [130]. Qi *et al*. [131] revealed that 17-DMAG decreased the remodeling of the aortic wall, angiogenesis, and inflammatory responses in Ang II-induced AAA. Therefore, enhanced tube formation by Ang II-treated ECs was significantly reversed by 17-DMAG in an angiogenesis experiment [131].

## 5. Clinical Application of Angiogenesis

As angiogenesis has been shown to be a pathological marker of AAA in both human and animal models, detecting angiogenesis-related molecules may be a new technique for evaluating AA/AD to overcome the current limitations of diagnostic and prognostic assessment. Duan *et al*. [132] conducted a meaningful gene analysis by screening key genes related to angiogenesis using random forests and established an AAA diagnostic model with these genes using an artificial neural network. The diagnostic model had an area under the receiver operating characteristic curve of 0.786. This study suggested that angiogenesis is closely related to AAA, and monitoring angiogenesis-related molecules may facilitate dynamically evaluating AAA progression [132]. Based on this, Shi *et al*. [133] used ^64^Cu-labeled anti-CD105 antibody Fab fragment to image angiogenesis-related molecules and processes in a mouse model. Notably, enhanced contrast was achieved, and a higher level of neovascularization was detected at the ruptured edge of the AA, indicating that the imaging of angiogenesis-related molecules and processes can assist in the diagnosis of AA and potentially monitoring of high-risk AA/AD [133].

## 6. Conclusions and Prospects 

Angiogenesis is regulated by several pro- and anti-angiogenic factors that induce cell proliferation, migration, tube formation, and new capillary formation in pre-existing blood vessels. Although angiogenesis is a physiological process that initiates vascular repair, many preclinical studies have found that anti-angiogenic therapy effectively improves AA/AD injury. Current evidence suggests that angiogenesis not only responds to changes after vascular injury but also causes vascular homeostasis disorder.

In fact, AA/AD are two diseases with different diagnostic criteria in clinical practice, and the vascular components of thoracic and abdominal aortas originate from different embryonic layers. Therefore, certain differences in the pathophysiology of AA/AD originate from the thoracic or abdominal aorta. However, this review focused on the pathological role and potential mechanisms of angiogenesis in both AA/AD because of the following considerations. First, AA is a potentially life-threatening condition because it places patients at a risk for AD and rupture. Second, the thoracic or abdominal aorta has the same three-layer structure, and vasa vasorum penetrates the media from the adventitia and plays a role in nutrient delivery, cell infiltration, and vascular repair, which is crucial for vascular homeostasis [4]. Therefore, excessive neovascularization probably involves both AA/AD. Third, AA/AD have similar modeling methods in the classic animal model, and the two diseases usually coexist in the same model [134]. For instance, Ang II subcutaneous infusion for ${\mathrm{ApoE}}^{-,/-}$ mice and Ang II combined with BAPN induce the occurrence and development of both AA/AD, whether in the thoracic or abdominal aorta [135]. However, previous studies have demonstrated that pathophysiological changes, such as endothelial dysfunction [136, 137], phenotype switching of VSMCs [138], ECM degradation [139], inflammation [139], and oxidative stress [140], lead to both AA/AD in animal models. Fourth, some gene mutations were validated as an important cause of familial and nonfamilial nonsyndromic AA/AD, such as those in *TGFBR1*, *ACTA2*, and *FBN1*, among others [141]. Finally, current evidence of angiogenesis between AA and AD is mutually argumentative and complementary; therefore, integrated evidence from AA/AD could provide a deeper understanding. Therefore, although the current evidence is insufficient, angiogenesis is probably involved in both AA/AD.

Notably, there are some difficulties to overcome in clinical practice before applying angiogenesis as an intervention target. First, solving the problem of anti-angiogenesis of the vasa vasorum could systematically affect the vascular ECs. For example, VEGF pathway inhibitors can effectively inhibit angiogenesis and induce endothelium-dependent vasodilatory dysfunction and activate the renin-angiotensin system. Second, angiogenesis is a repair reaction, and excessive inhibition of angiogenesis leads to tissue ischemia and hypoxia [15]. Therefore, the timing of anti-angiogenesis is important because it is difficult to control. Third, angiogenesis in AA/AD is complex, and it involves a very complex interactive network, including angiogenic factors and cells, such as VSMCs, fibroblasts, macrophages, mast cells, and neutrophils. However, a simple and effective anti-angiogenesis treatment for AA/AD remains uncertain to date.

Vascular adventitia remodeling and local microenvironment changes are most likely to induce angiogenesis from the adventitia vasa vasorum in the early stages of AA/AD. In addition, fibroblasts are the main cell components of the adventitia. In the early stages of pathological changes, the activation of fibroblasts may cause their differentiation into myofibroblasts, thereby enhancing contraction, migration, and proliferation, promoting the production of cytokines and chemokines, and leading to ECM remodeling [71]. In rheumatoid arthritis, cancer, and inflammatory bowel disease, fibroblasts have been shown to drive angiogenesis in tissues [142, 143]. However, in AA, it is suggested that fibroblasts are associated with hypoxia-related angiogenesis [70]. Therefore, the triggering of angiogenesis by fibroblasts in AA/AD may be a novel and potentially critical mechanism; however, it is necessary to demonstrate these hypotheses. In addition, cells associated with adventitia, such as perivascular adipocytes, also deserve attention. A previous study showed that adipose tissue triggers the growth of blood capillaries, and in turn, adipose tissue ECs promote pre-adipocyte proliferation [144]. In AAA, lipid storage is upregulated in the adventitia, and adipogenesis is associated with angiogenesis [70]; therefore, insights into the cellular and signaling mechanisms underlying adipose tissue related-angiogenesis may also have important implications. In conclusion, current research suggests that angiogenesis is a new therapeutic target for AA/AD; however, discovering the initiating factors of uncontrolled angiogenesis and avoiding the cardiovascular side effects of anti-angiogenic drugs are a crucial focus in the future.
